# Ireland's Prosperity Unexampled: Ireland for the Irish—With British Millions for Irish Cultivators!

**Published:** 1918-04-27

**Authors:** 


### THE CARE OF LUNATICS IN IRELAND.

### IRELAND'S PROSPERITY UNEXAMPLED.

### Ireland for the Irish—With British Millions for Irish Cultivators!

The Report of the Irish Inspectors of Lunatics for the year 1916 is just issued. It records, as the Report of the English Commissioners of the Board of Control reports, for the second time in fifty years, the first time being in the previous year, a diminution in the number of the insane officially known to the lunacy authorities. Whether this corresponds to an actual diminution of the number of insane persons in the population is not known. Probably it is, but it may not be. Everything has been dislocated by the war, and it may be that insane persons who would have been certified in pre-war conditions are now being withheld from certification. The friends of the insane are always reluctant to certify their insane relatives. The insane are themselves reluctant to be certified. It may be that in England and Scotland, at any rate, both are taking advantage of the confusion and absorption of attention in the war and all that pertains to it to avoid certification. But there is no reason why this should be so in Ireland. The war scarcely affects Ireland, which is now in a condition of unexampled prosperity, fattening on the necessities of the other parts of the kingdom, and contributing little in sacrifice to the war, nay, resenting with furious and even frenzied resentment the proposal that it should take its share of the joint burden and contribute its fair share of manhood to the protection of these realms.

### The Irish are a Much Cleverer People than the English (*Bernard Shaw.*)

Its mottoes are "Ireland for the Irish" and "English money for the Irish," and the Irish judiciously refuse to imperil that overflowing prosperity that has resulted from the purchase of Irish land by English millions, and the handing over of this land thus purchased to the Irish cultivators. If Englishmen and Scotchmen choose to sacrifice their money in this way for the benefit of Ireland, why should they not also sacrifice their lives for the protection of Ireland from the Boche and leave the Irish at peace to attend to their own business, which is selling the produce of their land, purchased with English and Scotch money, to the English and Scotch at war prices? This is the common-sense view, and the Irish, as that good specimen of the Irish, Mr. Bernard Shaw, is fond of telling us, are a much cleverer people than the English. There are times when we have had our doubts of the accuracy of others of Mr. Shaw's assertions, but we see no reason to doubt this one. What could be more clever than to shirk on to your stupid neighbour, who stupidly allows himself to be influenced by such stupid motives as honour, patriotism, duty, and so forth, the task of defending you, or than at the same time taking advantage of his stupidity and his previous generosity to make money out of him? Certainly Mr. Bernard Shaw is right about his fellow countrymen. They are much cleverer than we Britishers are.

Be the cause what it may, it is certain that the number of insane officially recorded in Ireland decreased in the year 1915 by 77, and in the year 1916 by 337, the decrease in 1915 being solely among the males, while the number of females actually increased, and in 1916 the decrease is in both sexes, but chiefly among the females.

### Irish Neglect of the Insane in Institutions.

If we pass from statistics to consider the general condition and accommodation provided by the Irish for their lunatics, we find still further reason to admire the superior ability of the Irish over the dense Englishman and the stupid Scotchman. On this side of St. George's Channel we lavish care upon those unfortunate members of our community who become afflicted with the most terrible calamity to which human beings are subject. We house them in palatial buildings, we see to it that they are well bedded, well clothed, well fed, that they have ample air space, ample opportunities for exercise, occupation, and recreation. How much better do the clever Irish manage their insane! All these things cost money, and tend to the prolongation of useless lives. The clever Irishman recognises this, and allows it its due weight in his treatment of the insane, while the stupid Scotchman and the dense Englishman blunder on and try to ameliorate the lot of the insane and to keep these useless persons alive. If the conditions reported by the Inspectors of Lunatics to exist in many Irish asylums were to obtain in any English or Scotch asylum, there would be such an outcry against the inhumanity of the managers as would sweep them out of their offices, even if it took an Act of Parliament to do it.

### Some of the Abuses Reported.

These are some of the conditions reported by the Irish Inspectors of Lunatics to the Lord-Lieutenant and the Lord Chancellor of Ireland. In the Antrim Asylum the overcrowding is somewhat less acute. At Ballinasloe the asylum continues to be greatly overcrowded, and "as usual" the list of casualties and cases of zymotic disease was a heavy one. At Castlebar there is great overcrowding, and the nursing staff is inadequate. At Clonmel the male building (? building accommodating male patients. Or are the buildings in Ireland of different sexes?) is quite unsuitable to the accommodation of the insane. At Youghal the inspectors suggested that the patients should be allowed some margarine or butter with their bread at breakfast. Surely a very unnecessary extravagance! At Ennis the overcrowding, so often referred to in previous reports, continues. The bathing and sanitary accommodation are in need of

**THE CARE OF LUNATICS IN IRELAND—(cont.).**

improvement, and the staff of attendants still remains inadequate. There is no suggestion that this inadequacy is due, as in England, to the call of the war. Such a suggestion would, indeed, be manifestly absurd. At Killarney there is *still* need for a more liberal supply of papers, magazines, and books. At Limerick there is a similar dearth. At Maryborough a number of the beds "require attention"; the condition of some of the single rooms is "not altogether satisfactory"; the airing courts are small, cheerless, and shut in by buildings. Monaghan Asylum is still greatly overcrowded. At Mullingar no steps have yet been taken to relieve the great overcrowding, and the floors of some of the single rooms are of concrete. At the Richmond Asylum, "as usual," there were a considerable number of casualties and of cases of zymotic disease. Portrane is still infested with rats. Sligo, in addition to being greatly overcrowded, stands in need of many improvements. The staff is inadequate, and the use of mechanical restraint excessive.

WHAT IS THE STATE OF WARDS IN IRISH WORKHOUSES?

If these are the conditions prevailing in the asylums, we may expect that the state of the wards of workhouses accommodating the insane is not better, nor do we find it better. Their accommodation, say the inspectors, in many cases left something to be desired, and in a certain number was distinctly bad. When even the inspectors admit that the accommodation was distinctly bad we may be sure that it was bad—very bad. In some cases, they say, there was a decided lack of cleanliness—lack of cleanliness is, we should suspect, a miosis—"and in one or two conditions were found which were nothing short of disgraceful." They must have been bad indeed to evoke such an expression from the mild and gentle inspectors. They did not enter into particulars, and we are left to conjecture where these disgraceful conditions prevail, and, generally, what they are; but on the latter point the inspectors afford us a tantalising glimpse. The sanitary accommodation consists in many cases, it appears, only of buckets in the dormitories and privies out of doors, while as to the bathing appliances, portable baths, and even tubs, are in very general use, and in some institutions there is no regular bathing at all. If we omitted the word "regular" we should probably not over-shoot the mark.

"IRELAND SEEMS TO BE DOING PRETTY WELL."

This is the official Report of the Inspectors of Lunatics appointed by the Government of Ireland to inspect and report upon the condition of the institutions in Ireland devoted to the care of the insane. We have quoted their very words, and we need not comment upon them further than to point out how thoroughly they justify Mr. Bernard Shaw's estimate of the superior cleverness of the Irish race. Ireland is a poor nation, it is true, but it has now attained a pitch of prosperity unexampled in its history, and baths are not so very expensive. Papers, magazines, and books cost more than they did before the war, but evidently the shortage of paper is not as great in Ireland as it is here, for the inspectors publish in their report the usual mass of futile statistical tables, which have been omitted, in order to save the cost of paper and printing, from the Reports of the English Commissioners. It is evident, therefore, that in the matter of paper, as well as in other respects, the hardships inflicted upon England by the war have not crossed St. George's Channel. For a down-trodden, persecuted, distressful country, Ireland seems to be doing pretty well, and it seems that we may now save for our own use some of the pity and solicitude that we have so long lavished upon it. It has not yet obtained Home Rule in everything, but it certainly succeeds in managing its own local affairs in its own way, and no doubt it is open to the Irish to tell us that how it manages its own affairs is no concern of ours. It is no concern of ours, but still we may hold our own private opinion.

